# Stress‐induced hyperphagia? Characterising the activity of the ghrelin axis in male rats with high anxiety behaviour

**DOI:** 10.1111/jne.70106

**Published:** 2025-11-06

**Authors:** Amanda K. E. Hornsby, Bradley Haywood‐Sheldrake, Katharina Gryksa, Andrea Havasi, Luke D. Roberts, Trevor Humby, Inga D. Neumann, Jeffrey S. Davies, Timothy Wells

**Affiliations:** ^1^ School of Biosciences Cardiff University Cardiff UK; ^2^ Department of Behavioural and Molecular Neurobiology University of Regensburg Regensburg Germany; ^3^ Institute of Life Science, School of Medicine Swansea University Swansea UK; ^4^ School of Psychology, Cardiff University Cardiff UK

**Keywords:** anxiety, caloric restriction, feeding patterns, ghrelin, neurogenesis

## Abstract

While evidence is emerging that the temporal pattern of feeding may influence anxiety, it is unclear to what extent anxiety may itself impact spontaneous feeding behaviour. To address this, we have quantified spontaneous feeding, ghrelin secretion and adult hippocampal neurogenesis (AHN) in male low (LAB) and high (HAB) anxiety‐behaviour rats. LAB and HAB rats showed the expected anxiogenic profile in the elevated plus‐maze, HAB rats avoiding the open arms entirely. A 16% reduction in total food intake in HAB rats (*p* = .017) was due to a 35% reduction in light phase food consumption (*p* = .004). However, there were no significant changes in the number or duration of individual feeding events, and the 24‐h feeding profile remained largely unaltered. Although basal circulating ghrelin was comparable in HAB and LAB rats, the 57% elevation in circulating ghrelin induced by a 24‐h fast in LAB rats (*p* = .022) was completely abolished in HAB rats. In comparison with adult LAB rats, the number of newborn neurones (BrdU^+^/NeuN^+^) in the dentate gyrus of HAB rats was elevated by 68% and 103% in the sub‐granular zone and granule cell layer, respectively (*p* = .0004 and *p* < .0001), these increases being observed across the rostro‐caudal extent of the hippocampus. In contrast, the number of newborn non‐neuronal (BrdU^+^/NeuN^−^) cells was unaltered. Thus, even in the context of the marked anxiety in HAB rats, mild hypophagia occurs without significant alteration in feeding patterns. Despite a blunting of fasting‐induced ghrelin release, elevated AHN suggests an appropriate feedback response to the increased anxiety‐related behaviour.

## 1 INTRODUCTION

Despite the common perception that feeding behaviour and anxiety are interrelated, the exact nature of that relationship remains poorly defined.

On the one hand, a growing body of evidence supports the notion that fasting is associated with improved mood and reduced anxiety.^1^, ^2^ For example, fasting with caloric restriction (CR) resulted in decreased tension, anger, confusion and mood disturbance in older men.^3^ Conversely, the hyperphagia condition Prader–Willi syndrome is commonly associated with heightened anxiety.^4^ More detailed knowledge of this relationship has emerged from preclinical studies, where acute CR^5^ and time‐restricted feeding^6^ have been reported to reduce anxiety in male and female^7^ rats, and elevate basal corticosterone levels without augmenting stress‐induced hypothalamo–pituitary–adrenal (HPA) axis activation.^8^ While the antidepressant impact of CR may be mediated in part by orexin,^9^ the anxiolytic impact may be dependent upon the activation of the growth hormone secretagogue receptor (GHSR) by the gastric hormone ghrelin,^10^, ^11^ potentially via augmentation of vagal sensory signals.^12^ Conversely, it is also evident that central ghrelin treatment may be anxiogenic.^13^, ^14^, ^15^

Evidence that anxiety influences feeding behaviour is even more equivocal. For example, several studies have linked early post‐natal stressors with the development of obesity (reviewed in^16^), and elevated anxiety was associated with increased caloric intake in women.^17^ Conversely, presurgical stress and anxiety failed to alter food intake or dietary selection in men.^18^ Again, in a preclinical context, while a number of rodent studies indicate that restraint stress reduces food intake (reviewed in^16^), neonatal maternal separation results in adolescent‐onset hyperphagia^19^; the reverse being observed when the offspring are exposed to an additional stressor.^20^

Adult hippocampal neurogenesis (AHN), especially in the caudal dentate gyrus (DG), is a key cellular correlate of reduced anxiety. It is accelerated by CR^21^ and ghrelin exposure.^22^ Indeed, deletion of GHSR, the receptor for ghrelin, inhibits CR‐induced augmentation of AHN and associated fear‐memory performance.^23^ Mice with impaired AHN show a marked increase in anxiety‐related behaviour,^24^ and although mice with augmented AHN show normal basal anxiety, glucocorticoid‐induced anxiety is attenuated.^25^ Thus, AHN is uniquely positioned to connect feeding behaviour with anxiety via the activity of ghrelin.

Given this evidence for a role of AHN in feeding‐induced modulation of anxiety, this study tests the obverse hypothesis, that altered AHN accompanies anxiety‐induced changes in feeding. We tested this hypothesis by quantifying AHN, ghrelin secretion and indices of spontaneous feeding behaviour in rats selectively bred for high (HAB) and low (LAB) anxiety‐like behaviour.^26^ HAB rats also display an initial reduction in hippocampal cell proliferation,^27^ with subsequent development of depressive‐like, social and cognitive behaviours.^28^

## 2 METHODS

### 2.1 Animals

The animal procedures reported here were performed at the University of Regensburg in accordance with international guidelines on the ethical use of animals, including the Guide for the Care and Use of Laboratory Animals by the National Institutes of Health, Bethesda, MD, USA, and the ARRIVE guidelines and were approved by the government of Unterfranken, Bavaria, Germany.

**FIGURE 1:**
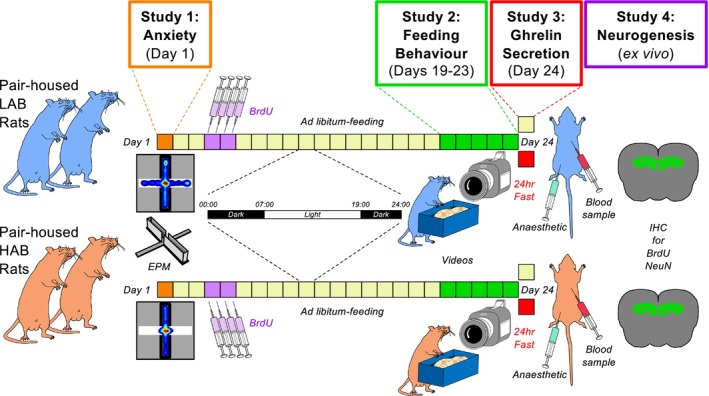
Experimental design to study the impact of high and low anxiety on feeding behaviour, ghrelin secretion and adult hippocampal neurogenesis (Studies 1–4). Anxiety‐like behaviour was assessed in pair‐housed 9‐week‐old male low‐anxiety behaviour (LAB) and high‐anxiety behaviour (HAB) rats in an elevated plus maze (EPM) (Study 1), followed by two twice‐daily i.p. injections of 5‐bromo‐2′‐deoxyuridine (BrdU). After 2 days of acclimatisation for crushed diet (days 19–20) food intake (total and light–dark phase) was estimated on Days 21 and 22 and feeding behaviour was videoed (Day 23). Rats remained either ad libitum‐fed or fasted for 24 h (Day 24) before being killed and blood samples and brain tissue collected for quantification of ghrelin and adult hippocampal neurogenesis.

Eight male HAB and eight male LAB rats (9–10 weeks old) were selected from an outbred Wistar rat colony according to their performance in an elevated plus maze (EPM) test, HAB rats being defined as those showing <5% of the test time in the open arm of the EPM and LAB rats >45% of the time in the EPM open arm.^26^, ^28^ These rats were kept pair‐housed in standard rat cages (55 × 35 × 20 cm) in the animal facilities of the University of Regensburg, Germany, under conditions of 12 h light/12 h dark (lights on at 07:00 h), with diet (regular diets grain‐based chow; Ssniff, Soest, Germany) and water available ad libitum. The overall experimental design is illustrated in Figure 1.

### 2.2 Study 1: Anxiety‐like behaviour in LAB and HAB rats

At 9 weeks of age, all HAB and LAB offspring were placed individually in an EPM for 5 min without prior handling, to assess basic levels of anxiety‐related behaviour. The time spent on and the number of entries into the open arms were recorded as indications of anxiety‐related behaviour, whereas the number of entries into the closed arms indicated locomotor activity.

### 2.3 Study 2: Spontaneous feeding behaviour in LAB and HAB rats

Given the anxiogenic effects of social isolation,^29^ single housing of HAB rats is not advisable^28^, ^30^ and, therefore, accurate measurement of individual food intake in metabolic cages is not achievable. To overcome this, pair‐housed animals received food via cage side‐mounted food hoppers containing crushed Ssniff chow 14 days after the last 5‐bromo‐2′‐deoxyuridine (BrdU) injection (Figure 1). After a further 2 days of acclimatisation, the food hoppers were refilled and weighed at 07:00 and 19:00 h (the beginning and end of the light phase) for two consecutive days. Individual total and light‐phase/dark‐phase food intake was estimated by dividing the amount of diet consumed by two.

To assess temporal feeding patterns, one rat in each cage received an identifying marker‐pen label on its head/body, and the cages were placed in front of a digital video recording camera for 24 h. Video recordings were analysed using EthoVision XT7 (Noldus Information Technology, Wageningen, the Netherlands) to quantify the number and duration of feeding events for each rat during the light and dark phases.

### 2.4 Study 3: Ghrelin and corticosterone secretion in LAB and HAB rats

To examine whether the observed differences in anxiety‐related behaviour and AHN between HAB and LAB rats may be related to altered ghrelin secretion, the groups of LAB and HAB rats were randomly divided into two equal sub‐groups (*n* = 4 per sub‐group). One sub‐group of each phenotype continued to be ad libitum‐fed, while the other sub‐groups were fasted for 24 h. All rats were asphyxiated with CO_2_ followed by rapid decapitation. Trunk blood was collected into EDTA‐coated tubes, centrifuged (2000*g*, 15 min, 4°C), 500 μL plasma collected and mixed with 50 μL 1 N HCl. While the addition of serine protease inhibitors is optimal for the protection of the octanoyl side chain, partial protection of acyl ghrelin can be achieved by acidification with HCl.^31^ Aliquots were stored at −80°C for subsequent determination of circulating ghrelin (total and acylated) and corticosterone by ELISA.

For the determination of circulating ghrelin, plasma samples were analysed by ELISA (Merck Rat/Mouse Ghrelin; total, EZRGRT‐91K, range: 160–10,000 pg/mL, intra‐assay variation [IAV]: 12%; active, EZRGRT‐90K, range: 32–1912 pg/mL, IAV: 19%) according to the manufacturer's instructions. ELISA plates were read on a CLARIOstar (BMG Labtech) plate reader.

In addition, circulating corticosterone was quantified in the same terminal samples via ELISA (ENZO Life Sciences, ADI‐900‐097, range: 0.032–20 ng/mL; IAV: 14%) following the manufacturers guidelines, as previously described.^32^ ELISA plates were read on a POLARstar Omega (BMG Labtech) plate reader.

### 2.5 Study 4: Quantification of adult hippocampal neurogenesis in LAB and HAB rats

To determine whether the elevated anxiety‐related behaviour measured in HAB rats was associated with reduced AHN, the rats were administered the thymidine analogue BrdU (50 mg/kg) via intraperitoneal injection, twice daily for 2 days (2 days after Study 1 and 13 days prior to the commencement of Study 2; Figure 1). After the decapitation (Study 3), brains were collected and post‐fixed in 4% paraformaldehyde for 1 day (4°C). The following day, brains were placed into 30% sucrose for 2–3 days and stored at −80°C until processed.

Brain sections (30 μm) were cut using a freezing stage microtome (MicroM HM450, Thermo Fisher Scientific) and collected in a 1:12 series into 24‐well plates containing phosphate buffered saline + 0.01% sodium azide storage solution. For the immunofluorescent detection of newborn mature neurones (BrdU^+^/NeuN^+^), brain sections were stained using a free‐floating method, as previously described.^23^ In brief, sections were stained for BrdU with a rat anti‐BrdU (MCA6144, Bio‐Rad) primary antibody and a corresponding fluorescent secondary antibody (Goat anti‐rat AlexaFluor488 [A11006, Thermo Fisher Scientific]), following which a primary antibody for mature granule cells (mouse anti‐NeuN [MAB377, Merck Millipore]) and an appropriate secondary antibody (Goat anti‐mouse AlexaFluor594 [A11005]) were applied. Subsequently, sections were treated with Hoechst as a nuclear counterstain.

Newborn neurons (BrdU^+^/NeuN^+^) and newborn non‐neuronal cells (BrdU^+^/NeuN^−^) in the DG of the hippocampus were quantified manually using a Leica DM 2700M Upright fluorescent microscope, under 40× magnification.

A cut‐off point of Bregma −2.70 mm was used to sub‐divide the data into rostral (rostral of Bregma −2.70 mm) and caudal (caudal of Bregma −2.70 mm) portions.

### 2.6 Statistics

Statistical comparison was performed by Student's unpaired *t*‐test (Studies 1, 2 [overall data and individual time points] and 4), and two‐way Analysis of variance (ANOVA) effects of phenotype and time (Study 2 feeding profiles) and phenotype and feeding status (Study 3) with Bonferroni multiple comparison post hoc test (ghrelin and corticosterone data; Study 3) using MS Excel 16.93 for Mac or Prism 10.6.0 for Mac respectively. Data shown are mean ± SEM.

## 3 RESULTS

### 3.1 Study 1: Anxiety‐like behaviour in LAB and HAB rats

**FIGURE 2:**
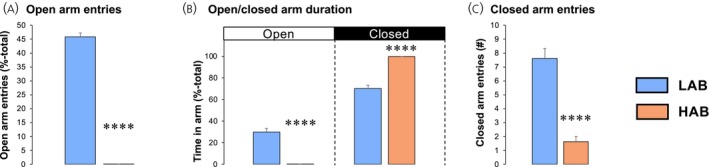
High‐anxiety behaviour (HAB) rats show complete aversion to an anxiogenic environment (Study 1). The number of open arm entries (A) and the proportion of total time spent in the open and closed arms (B) of low‐anxiety behaviour (LAB; blue, *n* = 8) and HAB (orange, *n* = 8) male rats placed in an elevated plus maze (EPM) for 5 min. The data shown are mean ± SEM, with statistical comparisons performed by Student's unpaired *t*‐test (****p < .0001 vs. LAB rats).

LAB rats displayed a 46% entry rate into the open arms of the EPM (Figure 2A), spending approximately 30% of the 5‐min test period in the open arms (Figure 2B). In contrast, none of the HAB rats entered the open arms during the test period (Figure 2A; *p* < .0001), spending 100% of the test period in the closed arms (Figure 2B; *p* < .0001). The number of closed arm entries, an index of locomotor activity, was lower in HAB rats (1.63 ± 0.38 entries/5 min) compared with LAB rats (7.63 ± 0.71 entries/5 min) (Figure 2C; *p* < .0001). Thus, the HAB rats used in this study present with a marked hyperanxiety phenotype.

### 3.2 Study 2: Spontaneous feeding behaviour in LAB and HAB rats

**FIGURE 3:**
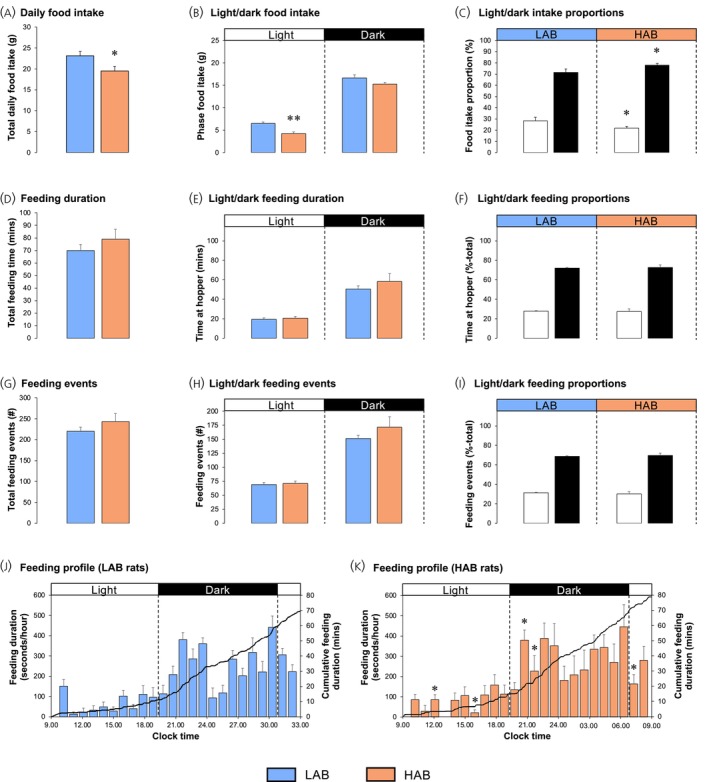
High‐anxiety behaviour (HAB) rats show mild hypophagia (Study 2). Estimation of 24‐h food intake (A), light‐ and dark‐phase food intake (B) and the proportion of food intake in the light and dark phases (C) in pair‐housed low‐anxiety behaviour (LAB; blue, *n* = 8) and HAB (orange, *n* = 8) male rats. In addition, the total duration (D) and number (G) of feeding events in individual rats, together with the duration (E) and number (H) of events in the light and dark phases and the proportion of these variables (F, I) in the light and dark phases are shown. The hourly consumption profiles of LAB (J) and HAB (K) rats are presented. The data shown are mean ± SEM, with statistical comparisons performed by Student's unpaired *t*‐test (*p < .05; **p < .01 vs. LAB rats).

Average total daily food intake in HAB rats was reduced by 16% (Figure 3A; *p* = .017 versus LAB rats). This was due to a 35% reduction in light phase food consumption (Figure 3B; *p* = .004), dark‐phase food intake not being significantly affected (Figure 3B; *p* = .060). As a result, HAB rats consumed proportionately more of their daily intake during the dark phase (6.5% higher) than LAB rats (Figure 3C; *p* = .040).

Analysis of the feeding videos of individual animals revealed that the mild hypophagia observed in HAB rats occurred in the absence of any significant changes in the duration of time spent at the food hopper (Figure 3D), or in the number of individual feeding events (Figure 3G). Similarly, there were no significant changes in these variables when separated into the light and dark phases (Figure 3E,H) and no change in the proportion of these events in the light and dark phases between LAB and HAB rats (Figure 3F,I). Using two‐way ANOVA to analyze temporal feeding patterns revealed a significant effect of time (*p* < .0001), no overall effect of phenotype (*p* = .146), and no significant interaction between these factors (*p* = .322), with both LAB and HAB rats showing the typical dark‐phase acceleration in feeding and a marked midnight nadir in the duration of feeding (Figure 3J,K). Hourly feeding duration in HAB rats was higher than in their LAB counterparts at 11:00–12:00 and 20:00–21:00 h and lower at 15:00–16:00, 21:00–22:00 and 07:00–08:00 h (Figure 3K; *p* < .05), indicating a faster commencement of dark‐phase feeding and a faster decline in light‐phase feeding.

It is interesting to note that, in the context of this hypophagia, body weight gain during the 48 h food monitoring period in HAB rats was 73% higher than in LAB rats (7.38 ± 0.86 g vs. 4.25 ± 1.46 g; *p* = .043; data not shown), but this was not maintained over the full 24‐day study period.

### 3.3 Study 3: Ghrelin and corticosterone secretion in LAB and HAB rats

**FIGURE 4:**
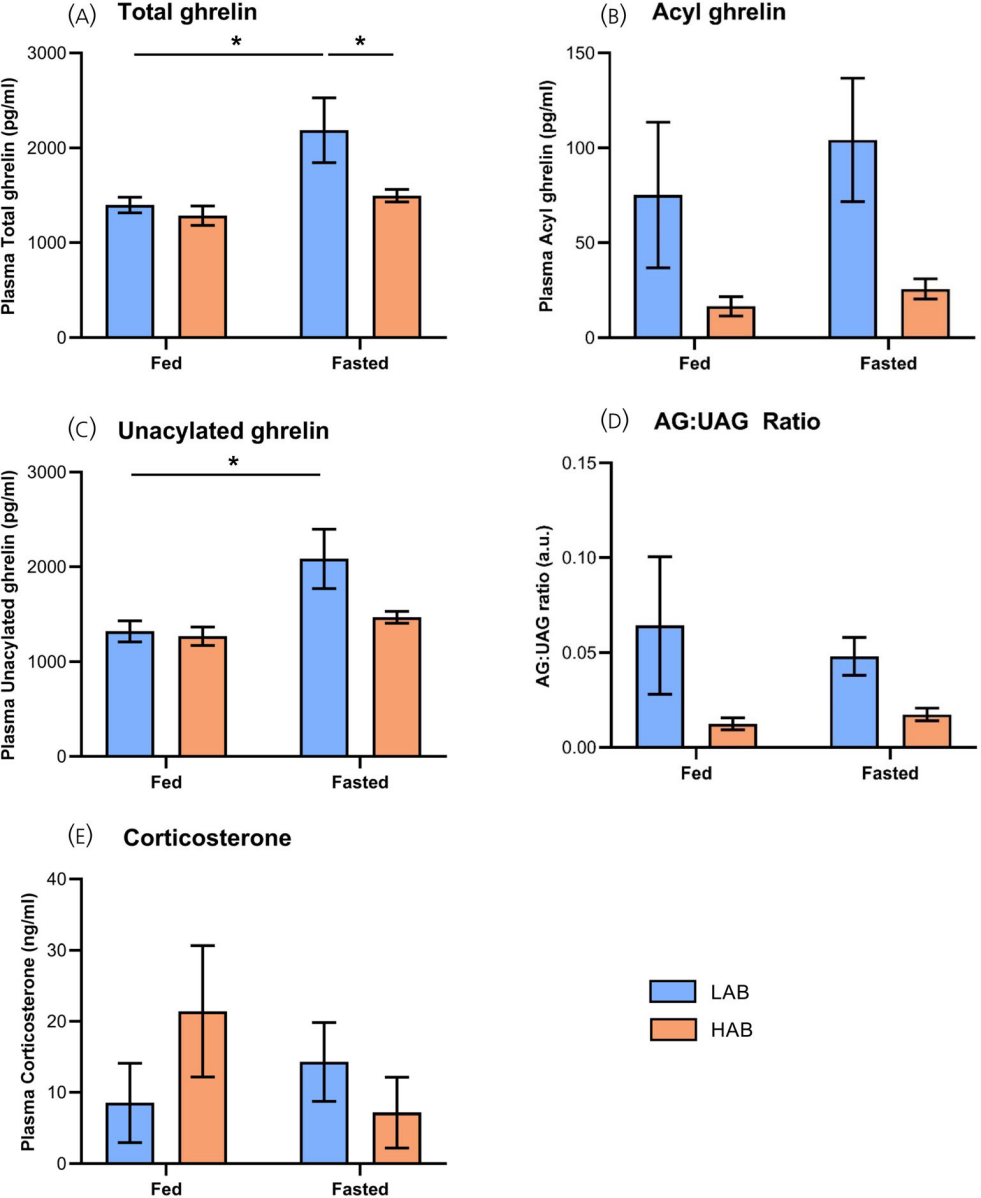
Fasting‐induced ghrelin secretion is abolished in high‐anxiety behaviour (HAB) rats (Study 3). Total (A), acylated (AG; B), unacylated (UAG; C) ghrelin, AG:UAG ratio (D) and corticosterone (E) in 12‐week old male low‐anxiety behaviour (LAB, blue) and HAB (orange) rats after ad libitum‐feeding or a 24‐h fast. Data shown are mean ± SEM (*n* = 4 for all groups), with statistical comparisons performed by 2‐way ANOVA and Bonferroni multiple comparison post hoc test (*p < .05).

Two‐way ANOVA revealed an effect of feeding status on total (*p* = .020) and unacylated ghrelin (UAG; *p* = .018). Although anxiety status influenced acylated ghrelin (AG) (*p* = .019), the impact on total and UAG was not significant (*p* = .052; .083). Although total and UAG levels in fed HAB rats were comparable to those in fed LAB rats (Figure 4A,C), mean circulating AG levels in fed HAB rats were only 22% of that in fed LAB rats (Figure 4B; *p* = .257 vs. fed LAB). As expected, a 24‐h fast elevated circulating total ghrelin in LAB rats by 57% (*p* = .022 vs. fed LAB; Figure 4A). This was due to a 58% elevation in UAG (*p* = .020; Figure 4C), mean circulating AG levels (Figure 4B) and the AG:UAG ratio (Figure 4D) being 138% and 75% of that in fully‐fed LAB rats (*p* = .871 and >.999). In contrast, this impact of fasting on the ghrelin system was abolished in HAB rats; total ghrelin (Figure 4A) and UAG (Figure 4C) levels in fasted HAB rats were 68% and 71% of those in fasted LAB rats (*p* = .044 and .060 vs. fasted LAB rats). Interestingly, circulating AG levels in fasted HAB rats were only 25% of that in fasted LAB rats (Figure 4B; *p* = .099 vs. fasted LAB rats), the AG:UAG ratio responding in parallel (Figure 4D; *p* = .546).

Although characterising HPA axis activity from terminal samples is impeded by the ultradian nature of corticosterone secretion, mean circulating corticosterone in fed HAB rats was 250% of that in fed LAB rats (*p* = .380; Figure 4E). However, mean corticosterone levels in fasted LAB rats were 168% of that in fed LAB rats (*p* > .999), whereas the mean corticosterone level in fasted HAB rats was only 34% of that in fed HAB rats (*p* = .301).

### 3.4 Study 4: Quantification of adult hippocampal neurogenesis in LAB and HAB rats

**FIGURE 5:**
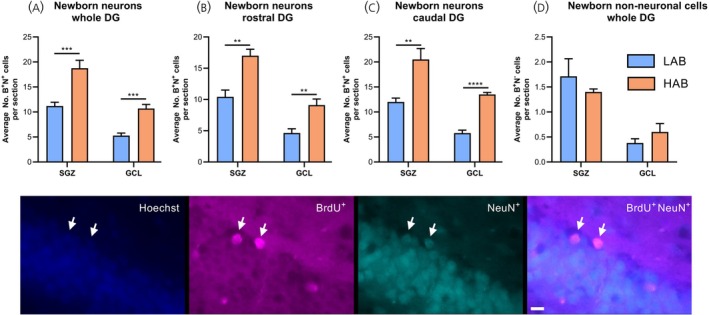
High‐anxiety behaviour (HAB) rats show elevated adult hippocampal neurogenesis (Study 4). Newborn neurones (BrdU^+^/NeuN^+^) in the sub‐granular zone (SGZ) and granule cell layer (GCL) of the whole (A) rostral (B) and caudal (C) dentate gyrus (DG) of 12‐week‐old male low‐anxiety behaviour (LAB, blue) and HAB (orange) rats, together with the number of newborn non‐neuronal (BrdU^+^/NeuN^−^) cells (D). Representative IHC photomicrographs of nuclei (Hoechst staining), BrdU‐positive (BrdU^+^), NeuN‐positive (NeuN^+^) and merged (BrdU^+^/NeuN^+^) images are presented. Data shown are mean ± SEM (*n* = 8 LAB rats, 6 HAB rats), with statistical comparisons performed by Student's unpaired *t*‐test (*p < .05; **p < .01 vs. LAB rats).

Quantification of AHN in the DG revealed that the number of newborn neurones (BrdU^+^/NeuN^+^) in the whole DG of HAB rats was elevated by 68% in the subgranular zone (SGZ; *p* = .0006; Figure 5A) and by 103% in the granule cell layer (GCL; *p* = .0001; Figure 5A). This augmentation of neurogenesis was replicated in both the rostral and caudal portions of the DG (*p* < .01; Figure 5B,C) and was observed in both the upper and lower blades (*p* < .0001; data not shown). This proliferation was specific to neuronal populations, as there were no parallel increases in the population of newborn non‐neuronal (BrdU^+^/NeuN^−^) cells (Figure 5D).

## 4 DISCUSSION

In the context of emerging evidence that elevations in ghrelin secretion induced by feeding patterns and CR promote AHN^33^ and reduce anxiety,^34^ we tested the obverse hypothesis, that elevated innate anxiety‐related behaviour disrupts temporal feeding patterns and is accompanied by reduced AHN. While the data we present from the HAB–LAB rat model do not support this hypothesis, elevated anxiety has a clear impact on the feeding‐ghrelin‐neurogenesis pathway. The hyper‐anxious HAB rats displayed mild hypophagia, but no overall change in the frequency or duration of feeding events. However, the blunted ghrelin response to fasting was accompanied by a marked augmentation of AHN in HAB rats.

The LAB and HAB rat lines, selectively bred for extremes in anxiety‐related behaviour,^28^ provide a unique opportunity to explore anxiogenic mechanisms and the impact of anxiety‐like behaviour on other physiological systems. As previously reported,^26^, ^28^ HAB rats displayed a marked anxiety phenotype in the current study, none of the rats entering the open arm of the EPM. This corroborates data obtained in alternative anxiety‐testing paradigms such as the open‐field test,^35^ the light–dark box^36^ and the modified hole board.^37^, ^38^ Similarly, the marked reduction in locomotor activity we observed has also previously been reported in this model.^39^

Given this behavioural phenotype, the HAB rats represent a most apposite model for characterising the impact of anxiety on feeding patterns. However, since single housing is particularly anxiogenic in these animals,^28^, ^30^ use of our automated feeding station^40^ was not feasible. We were, therefore, restricted to recording behavioural interactions with the food hopper in pair‐housed animals. Despite this limitation, we were surprised to find only subtle changes in feeding events between HAB and LAB rats, which remained broadly similar to the feeding parameters previously reported in male Sprague–Dawley rats in our feeding station.^40^ While both HAB and LAB rats showed a feeding surge at the commencement of the dark phase and a midnight nadir, the commencement of dark‐phase feeding and the decline in light‐phase feeding was faster in HAB rats. This accelerated transition was quantifiable despite the reduction in locomotor activity in HAB rats reported in both the current and previous studies.^38^, ^39^ In addition, an exaggeration in the diurnal feeding profile in HAB rats was evidenced by the greater proportion of food intake recorded in the dark phase and a corresponding reduction in the light phase. This reduction in light‐phase feeding occurred in the context of a doubling of circulating corticosterone and may reflect a chronic elevation in stress^41^ coupled with a potential aversion to light‐phase feeding. In this context, it is interesting to note that HAB rats have previously been reported to show a reduced preference for highly palatable high‐fat diets, potentially associated with lower hypothalamic urocortin2.^42^ Despite this amplification of the diurnal pattern, the reduction in light‐phase feeding was the primary determinant of the modest hypophagia seen in these animals.

In the context of hypophagia, the elevated weight gain over this period of the study was surprising. While this could result from the reduced metabolic demand of inactivity (as recorded in the EPM), altered ghrelin secretion, which defends body weight in the context of insufficient nutrient supply,^43^ is an alternative explanation. However, while total ghrelin levels did not differ between HAB and LAB rats under ad libitum conditions, the fasting‐induced ghrelin elevation in LAB rats was entirely suppressed in HAB rats. Interestingly, reduced circulating ghrelin has previously been reported in adolescent humans with avoidant/restrictive food intake disorder which is associated with an increased risk of anxiety.^44^ The accompanying reduction in the proportion of acylated ghrelin in HAB rats implies either a reduced ghrelin *O*‐acyl transferase activity or elevated action of de‐acylating enzymes. While the normal ghrelin levels during ad libitum feeding do not explain the faster onset of both dark phase feeding, and light phase satiety, the absence of a fasting‐induced ghrelin response accounts for the increased feeding latency previously reported in these rats following fasting.^45^ Nevertheless, the reduction in ghrelin activity reported here would usually result in reduced weight gain and a decline in other ghrelin‐dependent actions, including AHN.

Given that GHSR is highly expressed throughout the granule cell layer of the hippocampus,^23^, ^46^ the action of ghrelin at GHSR is essential for ghrelin‐induced AHN^23^, ^47^ and is opposed by UAG,^48^ it was surprising that AHN in HAB rats was elevated in both rostral and caudal portions. While these findings appear to contradict a previous report of reduced post‐natal neurogenesis in HAB rats,^27^ it corroborates the increase in hippocampal volume previously reported in adults.^28^ In the context of reduced post‐natal neurogenesis, heightened AHN may represent a developmental response to delayed neurogenesis and the anxiogenic environment in this model. Increased incorporation of newborn neurones into the rostral hippocampus is associated with enhanced spatial memory, while elevated neurogenesis in the caudal hippocampus is usually accompanied by reduced anxiety‐like behaviour.^25^, ^49^ Thus, while elevated AHN may contribute to the enhanced declarative memory performance in these animals,^50^ it appears that the presence of these newborn neurones is insufficient to ameliorate the impact of the anxiogenic signals.

A number of factors may promote the HAB rat phenotype, including altered HPA axis activity (for review see^28^). Although there is little difference in basal HPA axis activity between HAB and LAB rats, elevated adrenocorticotropic hormone (ACTH) and corticosterone responses to acute non‐social stressors in HAB rats have repeatedly been reported.^37^, ^51^ This hyperresponsiveness may result from overactivation of parvocellular paraventricular neurones,^35^ and overexpression of AVP that results from the presence of SNPs in the arginine vasopressin (AVP) promoter of HAB rats.^28^ Although the potential impact of fasting on the HPA axis in our study is partially obscured by low sample number and the pulsatile nature of corticosterone secretion,^52^ our data suggest that the impact of fasting on corticosterone elevation is reversed in HAB rats. Since ghrelin stimulates corticotropin releasing hormone (CRH) and AVP secretion^53^ and promotes ACTH secretion directly from the corticotrophs,^54^ the failure of fasting to stimulate ghrelin secretion in HAB rats may contribute to the reversal of the corticosterone response.

**FIGURE 6:**
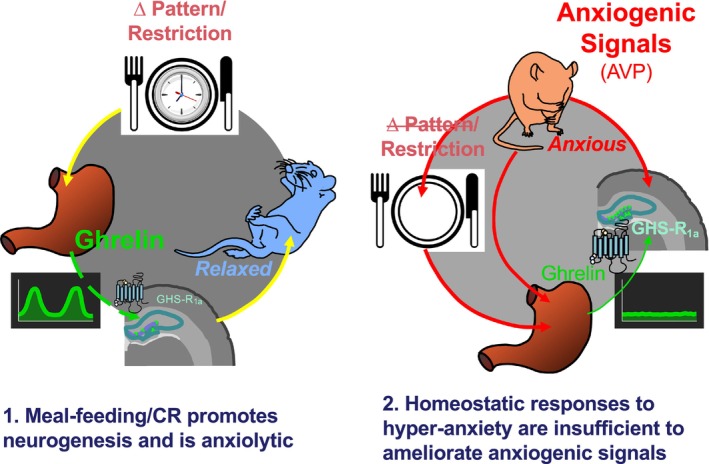
Homeostatic responses in hyper‐anxious rats are insufficient to ameliorate the impact of anxiogenic signals. Although altered feeding patterns and caloric restriction reduce anxiety in rats via the stimulation of ghrelin‐mediated hippocampal neurogenesis (1), anxious rats show mild hypophagia, blunted ghrelin responses and a homeostatic elevation in neurogenesis without any anxiolytic or anxiogenic changes in feeding pattern or any alleviation of anxiety.

In conclusion, while acute stressors suppress appetite and modify dietary selection through immediate neuroendocrine responses,^55^ we report that the chronic hyper‐anxiety phenotype in male HAB rats is not accompanied by either increased meal‐feeding or exacerbated grazing behaviour. However, the mild CR and elevated AHN potentially represent a homeostatic response to delayed hippocampal development and heightened anxiety (Figure 6). While elevated AHN may reflect augmented sensitivity to ghrelin, these responses are insufficient to overcome the powerful anxiogenic conditions arising from this trait‐selection breeding programme. We acknowledge that the restriction of our study to male rats represents a limitation, but we wished to address our hypothesis in the absence of the added complexities of higher AHN levels,^56^ enhanced anxiolytic response to ghrelin,^57^ altered anxiety behaviour across the oestrus cycle^58^ and reduced meal duration, especially at ovulation (see^59^) reported in females. In this context, whether the changes we report in males are more pronounced in females is yet to be determined. Nevertheless, while our data provide evidence of an exaggeration in the balance of diurnal feeding our study does not support the hypothesis that chronic anxiety modifies more dynamic parameters of temporal feeding behaviour.

## AUTHOR CONTRIBUTIONS

**Amanda K. E. Hornsby:** Conceptualization; investigation; methodology; writing – original draft; formal analysis; validation; writing – review and editing; data curation. **Bradley Haywood‐Sheldrake:** Investigation; methodology; formal analysis; data curation. **Katharina Gryksa:** Methodology; formal analysis; investigation; writing – review and editing; data curation. **Andrea Havasi:** Methodology; investigation. **Luke D. Roberts:** Formal analysis. **Trevor Humby:** Conceptualization; validation; formal analysis; writing – review and editing; supervision; funding acquisition; data curation. **Inga D. Neumann:** Conceptualization; writing – review and editing; supervision. **Jeffrey S. Davies:** Conceptualization; writing – review and editing; funding acquisition. **Timothy Wells:** Conceptualization; validation; writing – original draft; writing – review and editing; visualization; supervision; project administration; funding acquisition; data curation.

## CONFLICT OF INTEREST STATEMENT

The authors declare no conflicts of interest.

## ETHICS STATEMENT

The animal procedures reported here were performed at the University of Regensburg in accordance with the Guide for the Care and Use of Laboratory Animals by the National Institutes of Health, Bethesda, MD, USA, approved by the government of Unterfranken and performed according to international guidelines on the ethical use of animals and the ARRIVE guidelines.

## Data Availability

The data that support the findings of this study are available from the corresponding author upon reasonable request.
